# Tentacle Microelectrode Arrays Uncover Soft Boundary Neurons in Hippocampal CA1

**DOI:** 10.1002/advs.202401670

**Published:** 2024-06-03

**Authors:** Shiya Lv, Fan Mo, Zhaojie Xu, Yu Wang, Gucheng Yang, Meiqi Han, Luyi Jing, Wei Xu, Yiming Duan, Yaoyao Liu, Ming Li, Juntao Liu, Jinping Luo, Mixia Wang, Yilin Song, Yirong Wu, Xinxia Cai

**Affiliations:** ^1^ State Key Laboratory of Transducer Technology Aerospace Information Research Institute Chinese Academy of Sciences Beijing 100190 China; ^2^ University of Chinese Academy of Sciences Beijing 100049 China

**Keywords:** hippocampus, microelectrode arrays (MEAs), multi‐conductive layer, soft boundary neurons, spatial cognition neurons

## Abstract

Hippocampal CA1 neurons show intense firing at specific spatial locations, modulated by isolated landmarks. However, the impact of real‐world scene transitions on neuronal activity remains unclear. Moreover, long‐term neural recording during movement challenges device stability. Conventional rigid‐based electrodes cause inflammatory responses, restricting recording durations. Inspired by the jellyfish tentacles, the multi‐conductive layer ultra‐flexible microelectrode arrays (MEAs) are developed. The tentacle MEAs ensure stable recordings during movement, thereby enabling the discovery of soft boundary neurons. The soft boundary neurons demonstrate high‐frequency firing that aligns with the boundaries of scene transitions. Furthermore, the localization ability of soft boundary neurons improves with more scene transition boundaries, and their activity decreases when these boundaries are removed. The innovation of ultra‐flexible, high‐biocompatible tentacle MEAs improves the understanding of neural encoding in spatial cognition. They offer the potential for long‐term in vivo recording of neural information, facilitating breakthroughs in the understanding and application of brain spatial navigation mehanisms.

## Graphical abstract

How spatial cognition neurons encode scene transitions natural environments is an unknown field. Targeting this key scientific issue, an ultra‐flexible tentacle neural interface is developed. The high biocompatibility of tentacle microelectrode arrays ensures the long‐term stable detection. Using these electrodes, hippocampus neurons that fire intensely at the scene transitions are reported. This discovery will promote future brain‐controlled intelligent spatial navigation.

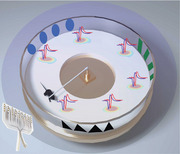

## 1 Introduction

The hippocampus is the brain's crucial area for constructing spatial cognitive maps, with its place cells encoding locations.^[^ ^1^ ^]^ The specific firing locations of place cells are known as place fields.^[^ ^2^, ^3^ ^]^ Interestingly, when the scene changes, the firing rate or place fields of place cells remap.^[^ ^4^ ^]^ Various factors, including visual cues,^[^ ^5^, ^6^ ^]^ odor cues,^[^ ^7^ ^]^ and proprioception,^[^ ^8^ ^]^ can trigger remapping in the spatial scene. The proposed explanatory hypotheses involve path integration,^[^ ^8^ ^]^ praxic navigation,^[^ ^9^ ^]^ and dead reckoning.^[^ ^10^ ^]^ However, scenes are often continuous in real‐world scenarios, such as commercial spaces and natural surroundings. Moreover, this is especially prevalent in virtual digital spaces where scene transitions are frequent.^[^ ^11^ ^]^ Therefore, whether place fields are influenced not only by isolated landmarks but also by scene transitions needs to be studied. If they do, does it imply that spatial cognition neurons respond to both isolated landmarks and scene transitions? It is essential because of the close connection between this spatial cognition research and future spatial navigation in virtual digital worlds.

Research on brain neural spatial cognition urgently requires electrodes capable of long‐term monitoring. However, rigid‐based electrodes induce severe inflammatory reactions.^[^ ^12^ ^]^ The relative drift between the brain and the electrode leads to chronic mechanical damage.^[^ ^13^ ^]^ This activates astrocytes and microglia, which form a neural glial sheath around the electrode.^[^ ^14^, ^15^ ^]^ Consequently, the signal‐to‐noise ratio (SNR) of recordings from rigid electrodes gradually deteriorates. Compared to Young's modulus of rigid electrodes, flexible electrodes ranging from 1 to 10 GPa^[^ ^16^ ^]^ offer a closer match to brain tissue.^[^ ^17^ ^]^ Therefore, polymer substrates, hydrogels,^[^ ^18^, ^19^ ^]^ and shape memory polymers^[^ ^20^, ^21^, ^22^, ^23^ ^]^ have been developed for flexible electrodes. Flexible electrodes based on polymer substrates include polyimide (PI),^[^ ^24^ ^]^ polydimethylsiloxane (PDMS),^[^ ^16^ ^]^ and SU‐8.^[^ ^25^ ^]^ PI, known for its corrosion resistance,^[^ ^26^ ^]^ along with SU‐8, which can be utilized in photolithography due to its photosensitive properties, enhances manufacturing flexibility. PDMS is durable in biological environments,^[^ ^27^ ^]^ but its thicker minimum profile (10 µm) limits some applications. Hydrogels are currently used for integrating microfilament electrodes with different functionalities, and their biocompatibility has been repeatedly verified. Shape memory polymers possess characteristics closely matched with brain tissue and can revert to their original shape upon external stimuli. However, the swelling coefficients of hydrogel and shape memory electrodes in terms of electrode thickness still need further reduction. In contrast, Parylene, an FDA‐approved material, combines corrosion and wear resistance with flexibility and insulating solid properties. Additionally, parylene can be deposited within a wide thickness range of 1–200 µm, which is easily controllable. These properties make it ideal for fabricating implantable flexible neural interfaces.^[^ ^28^, ^29^, ^30^, ^31^, ^32^, ^33^, ^34^, ^35^ ^]^ However, to enhance implantation rigidity, parylene‐based electrodes have been made thicker, which increases tissue damage.^[^ ^36^ ^]^ Moreover, capturing spikes with a high SNR still poses a challenge.^[^ ^37^ ^]^ Additionally, the electrodes signal acquisition capacity is limited, typically offering no more than 16 channels.^[^ ^38^ ^]^

To address the significant challenges mentioned above, we designed and fabricated the first multi‐conductive layer ultra‐flexible tentacle microelectrode arrays (MEAs) based on parylene. The tentacle MEA ensures increased detection throughput while reducing the implantation width by more than half, thus minimizing electrode‐induced damage to brain tissue. Subsequently, we implanted the flexible electrodes into seven mice. The implanted tentacle MEAs were used to study the localization function of the dorsal CA1 region of the mouse hippocampus during movement in scenes. We observed that nearly half of the 21 groups of neurons exhibited intense firing near the transition boundaries. Therefore, we propose naming these cells with such firing characteristics as “soft boundary neurons”. Furthermore, soft boundary neurons enhance their localization abilities as the number of boundaries in the scene increases. This biological phenomenon broadens the path for deciphering the mechanisms of spatial cognition encoding. Moreover, the self‐made, highly biocompatible tentacle MEAs open up broader possibilities for further exploration of spatial cognition.

## 2 Results

### 2.1 Morphology of the Ultra‐Flexible Tentacle MEAs

To achieve long‐term, precise, and stable detection in movement states using tentacle MEA, it is necessary to minimize the brain tissue damage and inflammatory responses caused by the electrodes.

**Figure 1:**
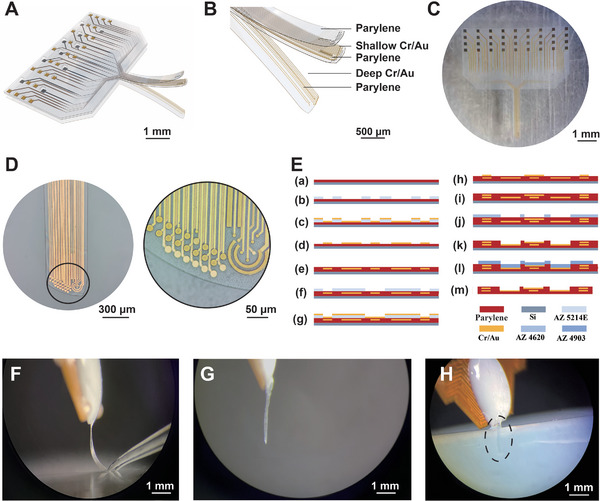
Morphology and fabrication of ultra‐flexible tentacle microelectrode arrays (MEAs). A) The overall concept of the multi‐conductive layers in tentacle MEA (scale bar: 1 mm). B) Local concept of the multi‐conductive layer at the tip of tentacle MEA (scale bar: 500 µm). C) Full view of tentacle MEA (scale bar: 1 mm). D) Demonstration of the deep and shallow conductive layers at the tip of tentacle MEA (scale bar: 300 µm), and details of the tip of the tentacle MEA in the black circle (scale bar: 50 µm). E) Fabrication process of tentacle MEA. a) Deposition of parylene onto a 4 in silicon wafer through chemical vapor deposition (CVD). b) Photolithographic patterning of the deep conductive layer. c) Deposition of the deep Cr/Au (30/200 nm) layer through physical vapor deposition (PVD). d) Removal of non‐targeted areas of the deep conductive layer. e) Deposition of the intermediate insulating layer of parylene through the second round of CVD. f) Photolithographic patterning of the shallow conductive layer. g) Deposition of the shallow Cr/Au (30/200 nm) layer through PVD. h) Lifting off non‐targeted areas of the shallow conductive layer. i) Deposition of the top insulating layer of parylene through the third round of CVD. j) Photolithographic patterning to expose microelectrodes, reference electrodes, bonding pads, and the outer contour of tentacle MEA. k) Etching of microelectrodes, reference electrodes, bonding pads, and the outer contour above parylene. l) Photolithographic patterning of the outer contour of tentacle MEA. m) Oxygen plasma etching to fully reveal the contour of tentacle MEA. F), Demonstration of the flexibility of tentacle MEA (scale bar: 1 mm). G), Tentacle MEA coated with PEG (scale bar: 1 mm). H), Implantation of tentacle MEA into 0.6% Agarose (scale bar: 1 mm).

Regarding material elasticity, a parylene substrate, in contrast to a rigid substrate, more closely aligns with the brain's Young's modulus. Furthermore, the surface design of the ultra‐flexible, 10 µm thick tentacle MEA is closely contoured to match the hippocampus (**Figure** 1D). The thickness of tentacle MEA is significantly reduced compared to the existing single‐conductive layer silicon‐based electrodes (≈30 µm)^[^ ^39^ ^]^ and flexible electrodes (≈15 µm).^[^ ^40^ ^]^

Regarding the multi‐conductive layer design, the electrode area, previously expanded laterally, is now stacked in an ultra‐thin structure. Therefore, the tentacle MEA reduces the implantation width by nearly half compared to single‐conductive layer electrodes while maintaining the same number of detection channels (Figure 1A–C). This reduction is aimed at minimizing implantation‐related damage and inflammatory responses. Additionally, the interleaved distribution of the deep and shallow conductive layers (Figure 1D), prevents overlap and eliminates signal cross‐talk caused by capacitive coupling.^[^ ^41^ ^]^

To ensure the single‐cell level detection resolution and stability of tentacle MEA, the following designs were implemented: microelectrodes of 20 and 10 µm were incorporated into the electrode (Figure 1D). This design fulfills the high spatial resolution electrophysiological detection requirements at the single‐cell level. Furthermore, dual open‐ring designs were implemented on their periphery because of the increased susceptibility of 10 µm microelectrodes to noise (Figure 1D), serving as a shielding mechanism.^[^ ^39^ ^]^ Neuronal wrapping aids in anchoring the electrode tip within brain tissue, further reducing relative motion between the electrode and the brain tissue.

### 2.2 Electrical Characterizations of the Tentacle MEAs

**Figure 2:**
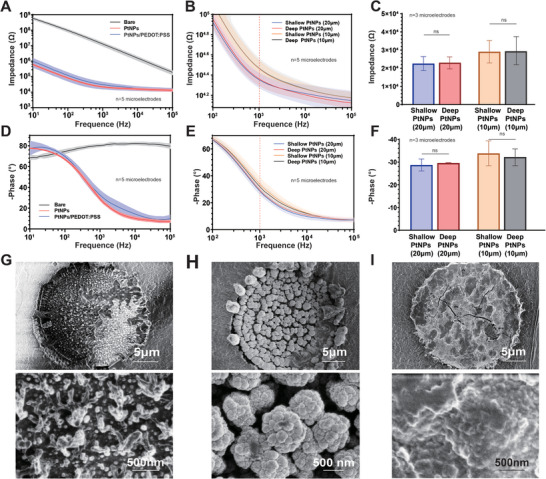
Electrical characterization of tentacle MEAs. A) Impedance of tentacle MEAs with the following conditions: bare surface, surface modified with platinum black nanoparticles (PtNPs), and surface modified with PtNPs/PEDOT. B) Impedance of tentacle MEA's surface after modification with PtNPs in a shallow conductive layer and a deep conductive layer. C) The impedance at the 20 and 10 µm microelectrodes in the deep and shallow conductive layers at 1 kHz. D) Phase of tentacle MEAs with the following conditions: bare surface, surface modified with platinum black nanoparticles (PtNPs), and surface modified with PtNPs/PEDOT. E) Phase of tentacle MEA's surface after modification with PtNPs in a shallow and deep conductive layer. F) The phase at the 20 and 10 µm microelectrodes in the deep and shallow conductive layers at 1 kHz. G) Scanning electron microscope image (SEM) of the bare surface of tentacle MEA (scale bar: 50 µm and 500 nm). H) SEM of tentacle MEA's surface after modification with PtNPs (scale bar: 50 µm and 500 nm).I) SEM of tentacle MEA's surface after modification with PtNPs/PEDOT:PSS (scale bar: 50 µm and 500 nm).The T‐test assessed statistical significance. ns, not significant. ^*^ *p* < 0.05, ^**^ *p* < 0.01; ^***^ *p* < 0.001, ^****^ *p* < 0.0001.

The electrical performance of tentacle MEA is the focus of detection. The surface of tentacle MEA is modified with platinum nanoparticles (PtNPs) and Poly(3,4‐ethylenedioxythiophene)‐poly(styrenesulfonate) (PEDOT:PSS), respectively. Only relatively smooth micro‐particles are on the bare electrode surface (**Figure** **2**G). In contrast, PtNPs form longitudinal nanoflower structures on the electrode surface (Figure 2H), increasing the contact area with neurons. Taking PtNPs modification as an example, when the microelectrodes are 10 µm, the impedance is 25 kΩ (Figure 2A,B), and the phase delay is ≈33° (Figure 2D,E). When the microelectrodes are 20 µm, the impedance is 23 kΩ, and the phase delay is ≈30°. After modifying PtNPs, the average normalized impedance difference at deep and shallow microelectrodes of 10 and 20 µm does not exceed 10%, and the average normalized phase difference does not exceed 8% (Figure 2C,F). Therefore, one of the factors influencing microelectrode impedance and phase delay is the electrode's surface area, and the distribution of conductive layers does not appear to have an impact.

Furthermore, to enhance the biocompatibility of the tentacle MEA, the microelectrode surfaces were further modified with PtNPs/PEDOT:PSS.^[^ ^42^ ^]^ Using the 20 µm site example, the impedance of PtNPs/PEDOT:PSS‐modified microelectrodes at 1 kHz is 30 kΩ (Figure 2A,B) with a phase delay of 35° (Figure 2D,E). Compared to the bare electrode, the impedance is reduced by nearly 3 orders of magnitude, and the phase delay decreases by 45°. In contrast to PEDOT:PSS modification partially fills the gaps in PtNPs, reducing surface roughness (Figure 2I). Compared to PtNPs, there is a slight increase in impedance and a slight increase in phase delay, but these changes are almost negligible.

In summary, the PtNPs/PEDOT:PSS modified on the ultra‐thin tentacle MEA with flexible multi‐conductive layers is designed to enable high‐fidelity, long‐term recording, and transmission of neural activity, laying the foundation for extended electrophysiological monitoring in moving states. Moreover, the textured dual‐open‐ring design also attracted hippocampal neurons for phagocytosis, facilitating neuronal clustering in that area.^[^ ^43^, ^44^ ^]^ In terms of electrical performance, it lays the foundation for the long‐term detection of neural electrophysiological signals during motion states.

### 2.3 Biocompatibility of the Ultra‐Flexible Tentacle MEAs

**Figure 3:**
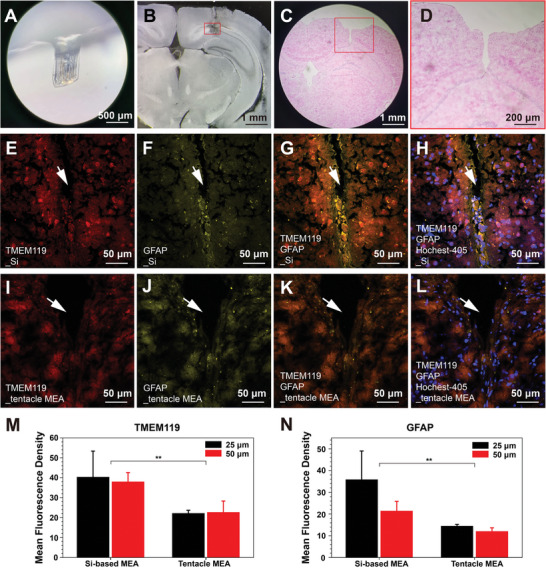
Implantation brain regions and biocompatibility of tentacle MEA. A) Surface condition of tentacle MEA 2 months post‐implantation (scale bar: 500 µm). B) The brain slice of tentacle MEA implanted in CA1 (The red box is the implantation location, scale bar: 1 mm). C) HE staining of mouse brain slice implanted with tentacle MEA (scale bar: 1 mm). D) An enlarged view of the implantation trajectory in Figure 3C (scale bar: 200 µm). E–H) Immunohistochemical staining images of a coronal brain slice after 2 months of a silicon‐based MEA implantation. The 10 µm‐thick slice was labeled for microglia (TMEM119, red), astrocytes (GFAP, yellow), and nuclei (Hochest‐405). I–L) Immunohistochemical staining images of a coronal brain slice after 2 months of a tentacle MEA implantation. The 10 µm‐thick slice was labeled for microglia (TMEM119, red), astrocytes (GFAP, yellow), and nuclei (Hochest‐405). M,N) The average fluorescence density of TMEM119 and GFAP outside the contour of the implanted Si‐based MEAs and tentacle MEAs at distances of 25 and 50 µm.The T‐test assessed statistical significance. ns, not significant. ^*^ *p* < 0.05, ^**^ *p* < 0.01; ^***^ *p* < 0.001, ^****^ *p* < 0.0001.

The 32‐channel silicon‐based MEA and tentacle MEA were respectively implanted in the CA1 of the dorsal hippocampus of mice. In spike recordings, the typical SNR is >10, and the SNR remains relatively stable over a month (Figure S3, Supporting Information). Two months after implantation, the flexible electrode was removed without any blood clots attached and with its surface intact (**Figure** **3**A). Brain tissue sections from different mice showed that the tentacle MEA was accurately implanted, and brain tissue sections stained with Hematoxylin and Eosin (HE; Figure 3B–D).

Immunofluorescence staining of microglia and astrocytes was utilized to validate the biocompatibility of the tentacle MEA. Microglia, the primary immune cells of the central nervous system, are activated and migrate to the injured area upon central nervous system injury, releasing inflammatory factors and phagocytosing pathogens and dead cells.^[^ ^45^ ^]^ Astrocytes, serving as supportive cells, also become activated in inflammatory responses and release inflammatory factors. Both cell types participate in inflammatory reactions and influence neuronal activity.^[^ ^46^ ^]^ Therefore, the degree of aggregation of microglia and astrocytes is used to assess the inflammatory response induced by the implantation of silicon‐based and flexible electrodes.

Around silicon‐based electrodes, microglia and astrocytes were observed to envelop the area tightly (Figure 3E–H). In contrast, the accumulation of microglia and astrocytes around tentacle MEA was less pronounced (Figure 3I–L). Analyze the mean immunofluorescence density of TMEM119 and GFAP within 25 and 50 µm outward from the contour of the tentacle MEA implantation (Figure 3M,N). Analyze the average immunofluorescence density of TMEM119 and GFAP within 25 and 50 µm outward from the contour of the electrode implantation. This shows that the reduced mechanical stimulation from the flexible electrodes significantly decreases the inflammatory response in the surrounding tissue in the later stages post‐implantation.

### 2.4 Presumption of Soft Boundary Neurons

**Figure 4:**
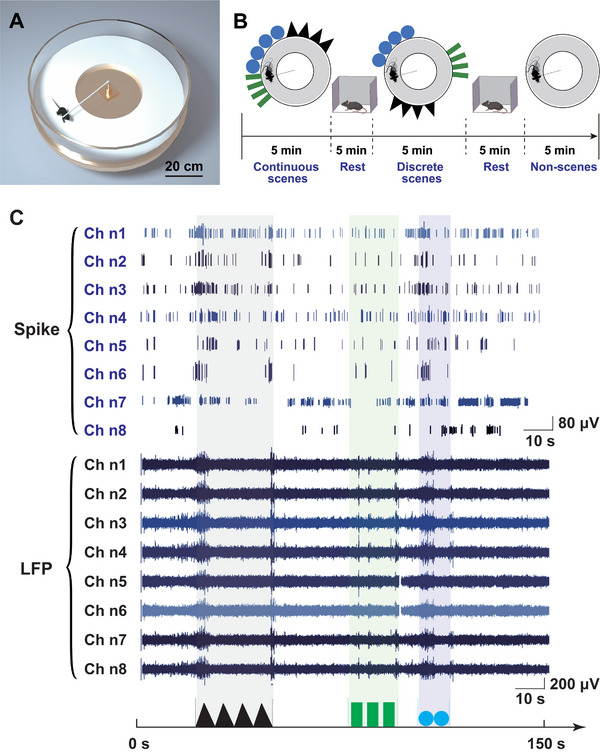
Electrophysiological recording of presumed soft boundary neurons in continuous scenes (CS), discrete scenes (DS), and non‐scenes (NS). A) Circular open field construction. B) Three scenes in which the mice ran, along with the training time and sequence. The mouse underwent a series of signal tests, running for 5 min in each CS (continuous black triangles, blue circles, and green rectangles), DS (discrete black triangles, green rectangles, and blue circles), and NS scenes. Between these trials, it was given a 5 min rest period in a cage. C) Electrophysiological and LFP signals detected during passage through DS, intense spikes and prominent LFP activity coincide with scene transition boundaries (real‐world discrete scenarios from 0 to 150 s) Electrophysiological and LFP signals detected during passage through DS, intense spikes and prominent LFP activity coincide with scene transition boundaries (real‐world discrete scenarios from 0 to 150 s).

To simulate continuous scenes, we created sets of cue cards, each extending over a length more than five times the mouse's body length. These cue cards included continuous black triangles, blue circles, and green rectangles. Additionally, to ensure consistent visual exposure, the mouse's head was immobilized with a rotated crossbar, allowing them to run along the edge in a directed manner (**Figure** **4**A).

In the first trial, the mice ran through the continuous scenes (CS), repeatedly passing the black triangles, blue circles, and green rectangles (Figure 4B, left). After 5 min, the mouse was removed and placed in a cage to rest for another 5 min. In the second trial, the CS was modified to a discrete scene (DS) layout, with the black triangles, blue circles, and green rectangles evenly distributed along the circular blank walls (Figure 4B, middle). After the trial, also allow a 5 min rest. In the third trial, all scenes were removed, leaving a non‐scene (NS) (Figure 4B, right). In the preliminary analysis of spike and LFP signals, it was found that some detected neurons exhibited intense firing at the boundaries of scene transitions (Figure 4C).

The increasing firing rate at scene transition boundaries may relate to specific features of scene changes, such as altered visual stimuli, new spatial settings, or other perceptual shifts. This pattern likely reflects the brain's segmentation of continuous visual information into distinct scene units for more efficient processing and memory.

### 2.5 Identification of Soft Boundary Neuron Traits

**Figure 5:**
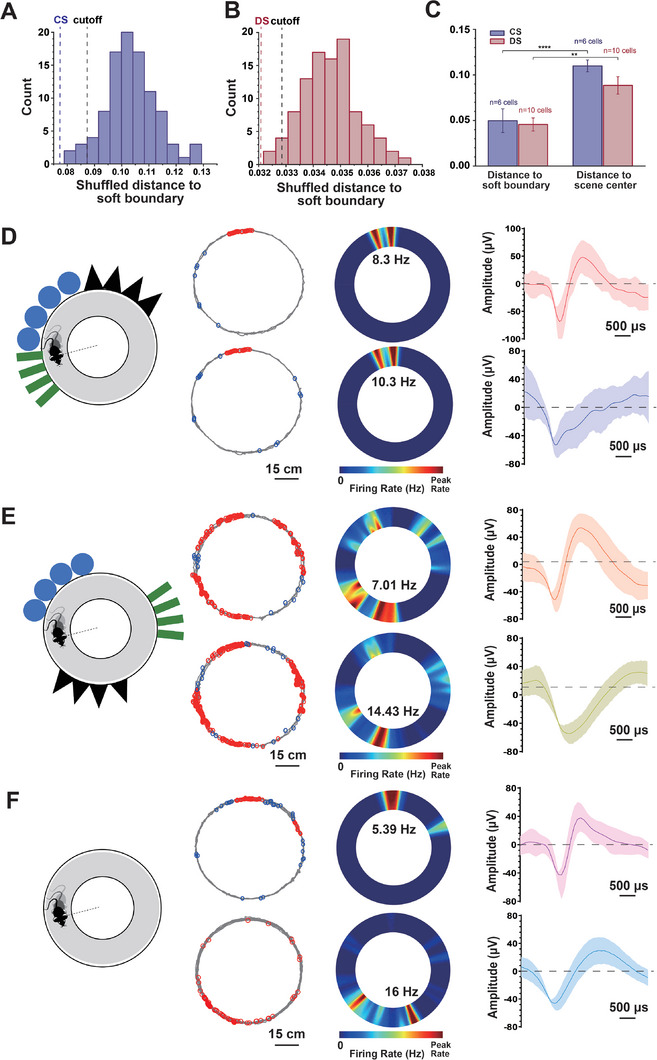
Identification and anchoring characteristics of soft boundary neurons. A) The distribution of distance to soft boundaries from 100 shuffled data of the soft boundary neurons in CS. The purple line indicates the actual distance to soft boundaries of the soft boundary neuron, and the black line indicates the 5_th_ largest distance to soft boundaries of 100 shuffled data. B) The distance distribution to soft boundaries from 100 shuffled data of the soft boundary neurons in DS. The red line indicates the actual distance to the soft boundaries of the soft boundary neuron, and the black line indicates the 5_th_ largest distance to soft boundaries of 100 shuffled data. C) Normalized average distance of soft boundary neurons firing locations to scene boundaries and center in CS and DS. D) Left: The CS setting for mice; Middle: Mouse's running trajectory (red circles indicate firing locations, scale bar: 15 cm) and spatial firing rate heatmap; Right: Spike waveforms of firing soft boundary neurons (scale bar: 500 µs). E) Left: The DS setting for mice. Middle: Mouse's running trajectory (red circles indicate firing locations, scale bar: 15 cm) and spatial firing rate heatmap. Right: Spike waveforms of firing soft boundary neurons (scale bar: 500 µs). F) Left: The NS setting for mice; Middle: Mouse's running trajectory (red circles indicate firing locations, scale bar: 15 cm) and spatial firing rate heatmap; Right: Spike waveforms of firing soft boundary neurons (scale bar: 500 µs).The T‐test assessed statistical significance. ns, not significant. ^*^ *p* < 0.05, ^**^ *p* < 0.01; ^***^ *p* < 0.001, ^****^ *p* < 0.0001.

In this context, the transitions between scenes are called “soft boundaries.” To further verify that the detected neurons specifically respond to boundary locations, we performed 100 shuffles of the spikes relative to their locations.^[^ ^47^, ^48^ ^]^ A neuron was defined as a soft boundary neuron if its proximity to the boundaries fell within the lowest 5% percentile in the shuffled data distribution (**Figure** **5**A,B). Moreover, compared to the scene center, the firing fields of soft boundary neurons are closer to the scene boundaries, with their normalized average distance within 5%. This distance is half that between the firing fields of soft boundary neurons and the scene centers (Figure 5C).

Three sets of scenes were constructed to sequentially verify the response of soft boundary neurons to soft boundaries. In the CS setup, featuring sequences of black triangles, blue circles, and green rectangles (Figure 5D, left), ≈30% of neurons exhibited intense firing at the soft boundaries between continuous black triangles and continuous blue circles (Figure 5D, middle). Among them, soft boundary neurons' spatial firing rate heat maps can reach 8.3 and 10.3 Hz (Figure 5D, middle motion trajectory and firing heat map). The peak‐to‐trough value of the spike waveform is less than 160 µV (Figure 5D, right).

In the DS setup (Figure 5E, left), the scene locations were separated, and the cue card placement order was reversed to further confirm the influence of soft boundaries on neuronal modulation. Nearly half of the neurons showed concentrated firing activity localized at the soft boundaries. The soft boundary neurons' spatial firing rate heat maps showed rates up to 7.01 and 14.43 Hz (Figure 5E, middle motion trajectory and firing heat map). The peak‐to‐trough value of the spike waveform is less than 160 µV (Figure 5E, right).

All scenes were removed in the NS (non‐scene) setup (Figure 5F, left). The number of firing fields of soft boundary neurons decreased, but they still converged at one or both of the boundaries from the DS scene, with spatial firing rates of 5.39 and 17.5 Hz (Figure 5F, middle motion trajectory and firing heat map). The peak‐to‐trough value of the spike waveform is less than 160 µV (Figure 5F, right).

Notably, the maximum firing locations are concentrated at the junction between the black triangles and the white space. Neurons appear to replay previous scenes, yet the replay's location slightly shifts and the corresponding number of soft boundaries decreases.

In summary, we discovered that specific neurons were primarily firing at the boundaries of scene transitions, so we named them “soft boundary neurons”. Once scenes are separated, a soft boundary neuron's firing field may also undergo a following expansion, particularly sensitive to the black triangle's scene boundary. These findings show that scene transitions can regulate the firing fields of spatial cognitive neurons. Furthermore, as scene transitions change, the place fields can expand. When there are no apparent scene transitions, the place fields re‐concentrate.

### 2.6 Impact of Soft Boundaries on Neural Dynamics

We observed that, in contrast to isolated visual landmarks,^[^ ^5^, ^6^ ^]^ soft boundaries can also positively impact the localization abilities of parts of neurons.^[^ ^49^ ^]^ This effect is evident in both temporal rate coding and spatial rate coding.

**Figure 6:**
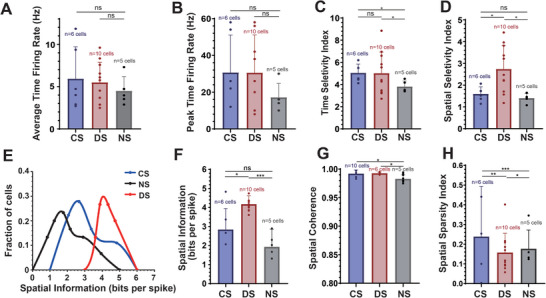
Impact of soft boundaries on neural dynamics. A) Average time firing rate of soft boundary neurons in CS, DS, and NS. B) Peak time firing rate of soft boundary neurons in CS, DS, and NS. C) Time selectivity of soft boundary neurons in CS, DS, and NS. D) Spatial selectivity of soft boundary neurons in CS, DS, and NS. E) Spatial information distribution of soft boundary neurons in CS, DS, and NS. F) Spatial information of soft boundary neurons in CS, DS, and NS; G) Spatial coherence of soft boundary neurons in CS, DS, and NS. H) Spatial information of soft boundary neurons in CS, DS, and NS. The T‐test assessed statistical significance. ns, not significant. ^*^ *p* < 0.05, ^**^ *p* < 0.01; ^***^ *p* < 0.001, ^****^ *p* < 0.0001.

Regarding the firing rate, the firing rate of soft boundary neurons remains almost unchanged compared to NS (**Figure** **6**A,B). The average temporal firing rates in CS, DS, and NS are 5.93, 5.5, and 4.5 Hz, respectively (Figure 6A). The average peak firing rates are 30.5, 30.7, and 17.2 Hz, respectively (Figure 6B).

Time selectivity and space selectivity were employed to compare further the localization abilities of soft boundary neurons in CS and DS.^[^ ^6^ ^]^ Time selectivity evaluates the response speed of soft boundary neurons to soft boundaries. In both CS and DS, the response speed of soft boundary neurons was similar, but in the non‐scene recall phase, their response speed notably decreased (Figure 6C). Space selectivity measures the degree of soft boundary neurons' response to specific areas. As the scene transitioned from CS to DS, the number of soft boundaries increased from 4 to 6, enhancing the response to spatial areas; this selectivity regressed upon removing the scene. However, there was no significant difference in space selectivity compared to NS and CS (Figure 6D).

Furthermore, we observed that the increases in time firing rates, time selectivity, and space selectivity are shown in the elevation of spatial information (Figure 6E). The spatial information shifts rightward with an increase in the number of boundaries. In DS, compared to CS, soft boundary neurons need to anchor to more boundaries, decoding a richer array of spatial locations (Figure 6F). However, there is still a discrepancy between spatial information content and the number of boundaries, showing that not all soft boundaries may be fully decoded. Moreover, in the NS scene, soft boundary neurons still map to the boundary conditions of DS. Hence, there is no significant difference from the scene with fewer boundaries in CS.

Soft boundary neurons specifically respond to boundaries, but the stability and distribution of their responses need to be quantified. Therefore, spatial coherence and spatial sparsity were calculated. In all three scenarios, soft boundary neurons exhibited high spatial coherence, indicating that their firing activity is clear and continuous, and they can stably respond to changes in location as the animal moves, with a lower probability of random activation or silence (Figure 6G). Spatial sparsity was used to describe the distribution characteristics of soft boundary neurons. In CS, with fewer boundaries, neuron anchoring is more concentrated, resulting in an average spatial sparsity of 0.24; in DS, the number of boundaries increases, spreading the firing fields and reducing sparsity to 0.16. In NS, sparsity slightly increases with a decrease in the number of firing fields (Figure 6H).

In summary, soft boundary neurons can stably anchor to scene boundaries. Moreover, as boundaries are updated and more complex, soft boundary neurons' localization ability also adjusts accordingly.

## 3 Discussion

In this study, we pioneered the application of a custom‐engineered tentacle MEA, specifically designed for long‐term electrophysiological recordings in mice during movement. The innovation of the tentacle MEA lies in its pioneering stack of ultra‐thin, multi‐conductive layers on a parylene substrate for detection. The tentacle MEA stands out for its exceptional mechanical compatibility with brain tissue, a critical advancement demonstrated to minimize electrode‐induced damage significantly. This approach not only preserves the integrity of the brain tissue but also markedly extends the electrodes' effective recording period.^[^ ^16^ ^]^ Currently, tentacle MEA focuses on concentrated detection in the hippocampal CA1 region, suitable for the high‐density neuron distribution in the hippocampal CA1 pyramidal layer. In the future, based on the morphology of the hippocampal region, increasing the number of electrode shanks to enhance channels can be attempted, aiming for synchronous and high‐density detection across multiple sub‐regions within the hippocampus.^[^ ^29^, ^30^, ^31^, ^32^, ^33^, ^34^, ^35^ ^]^ Moreover, implanting electrodes coated with PEG can be further improved using tungsten wire guidance to reduce implantation damage. We have observed persistent recordings from multiple neurons in mice for up to 1 month. The absence of further recordings was because of the termination of the experiment, not electrode failure, showing the possibility of further extending recording durations. Additionally, the tentacle MEA was used to detect the electrophysiological activity of hippocampal CA1 neurons in mice. Furthermore, suppose the implantation length of the tentacle MEA is extended to 6 cm in the future. It may be suitable for deep brain signal detection in primates, laying a solid foundation for long‐term free‐moving primate research. In terms of substrates, hydrogels and shape memory electrodes are promising future directions.^[^ ^18^, ^19^, ^20^, ^21^, ^22^, ^23^ ^]^ Hydrogels are repeatedly confirmed for their biocompatibility, and shape memory polymers return to their original shape with specific stimuli, facilitating easier implantation of flexible electrodes without rigid tools or surface modifications. However, material expansion should be considered with both materials to prevent tissue damage. In exploring spatial cognition in mice, our study can be contrasted with previous research on the relevance of visual stimuli to the hippocampus.^[^ ^5^, ^8^, ^49^ ^]^ However, the critical difference is that visual stimuli are not isolated local landmarks^[^ ^50^ ^]^ but rather scenes that are more than five times the length of a mouse, aiming to simulate changes in natural scenes. These scenes include both color and shape variations. Previous studies have shown that place cells exhibit significantly increased firing when repeatedly passing local landmarks, even before encountering the landmarks.^[^ ^51^ ^]^ Therefore, isolated local landmarks can induce place fields.^[^ ^52^ ^]^ However, this raises the question of whether changing boundaries in scenes (soft boundaries) can also act as visual stimuli, causing the anchoring of spatial cognition neurons. Consequently, three scenes, CS, DS, and NS, were set up, corresponding to 4, 6, and 0 soft boundaries, respectively. We found that the firing of some CA1 neurons, termed “soft boundary neurons,” was more pronounced at scene transitions.

We still require more detailed metrics to comprehensively assess the impact of scene transition on the spatial coding of soft boundary neurons. Therefore, we further characterized it using metrics such as time firing rate, time selectivity, spatial selectivity, spatial information, spatial coherence, and spatial sparsity. When animals transition from CS to DS, there is an increase in spatial selectivity, an elevation in spatial information content, and a reduction in spatial sparsity. This indicates that when animals are exposed to an entirely new scene, spatial cognitive neuron firing forms new representations, and independent maps are established for this new scene, consistent with previous findings.^[^ ^53^ ^]^ Moreover, in the NS, neurons appear to replay the previous scene. However, it is evident that the replayed locations are shifted, and the number of firing fields is reduced.

This work shows that first, scene transitions trigger neuronal encoding. The question then is whether the neuronal encoding of the two is consistent. Second, more boundaries correspond to more stable selectivity and pronounced firing; we consider whether spatial cognition neurons adopt an efficient and energy‐saving coding strategy. Lastly, we consider whether, in the NS scene, neurons fire intensely at the locations corresponding to the position of the previous scene due to recalling and encoding the previous scene.

## 4 Experimental Section

### Subjects

Seven female mice (C57BL/6N, 8–12 weeks, 22–25 g; Charles River Laboratory Animal Technology Co, Ltd) were recorded for the experiments. They were housed at 20 °C with a 12 h light: 12 h dark reversed light cycle. All animal care and procedures complied with the Institutional Animal Care and Use Committee at the Aerospace Information Research Institute, Chinese Academy of Science (AIRCAS), and experimental procedures were approved by the Beijing Association on Laboratory Animal Care. The approval number is AIRCAS‐002.

### Design and Fabrication of the Tentacle MEAs

The tentacle MEAs feature a multi‐conductive layer design (Figure 1A,B,D) aimed at minimizing the width of the implantation needle to mitigate neuronal damage during the implantation. The total thickness of the tentacle MEAs was 10 µm. The implantation needle was 3.5 mm long and 450 µm wide, with 32 microelectrodes concentrated at its tip (Figure 1D). These microelectrodes had diameters of 20 and 10 µm and were connected by 5 µm‐wide conductive lines at the rear. Among them, the 10 µm‐sized microelectrodes had a smaller contact area with neurons, leading to a lower SNR. Therefore, they were positioned within concentric shielding rings connected to the ground (G) to reduce noise interference. Two concentric shielding rings with inner diameters of 110 and 70 µm and a width of 10 µm were used. Furthermore, two larger G electrodes were positioned between microelectrodes as reference potentials for precise electrophysiological signal detection. These reference electrodes measured 15 × 300 µm and 10 × 300 µm. The tentacle MEA includes a 4 × 9 array of 200 × 200 µm bonding pad.

Similar to what was previously reported,^[^ ^38^ ^]^ the tentacle MEA was manufactured using Micro‐Electro‐Mechanical System technology (MEMS), following these steps:

1. A 4 in silicon wafer was cleaned, and a 6 µm substrate layer of parylene was deposited onto the silicon wafer through chemical vapor deposition (CVD) (Figure 1E(a)).
2. The initial photolithography process outlined the shape of the conductor layer (Figure 1E(b)).
3. The deep Cr/Au (30/200 nm) layer was structured through physical vapor deposition (PVD) and lift‐off. Chromium (Cr) was applied to enhance adhesion between the conductive layer and the substrate layer (Figure 1E(c,d)).
4. A 2 µm intermediate insulating layer of parylene was deposited on top through CVD (Figure 1E(e)).
5. A second photolithography step delineated the shape of the shallow conductor layer (Figure 1E(f)).
6. The shallow Cr/Au (30/200 nm) layer was formed via PVD and lift‐off (Figure 1E(g,h)).
7. A further 2 µm top insulating layer of Parylene was deposited to insulate the deep conductive layer through CVD (Figure 1E(i)).
8. In the third photolithography step, microelectrodes, reference electrodes, bonding pads, and the outer contour of the tentacle MEAs were exposed (Figure 1E(j,k)).
9. The final photolithography step utilized oxygen plasma etching to fully reveal the tentacle MEAs contour (100 W 10 min) (Figure 1E(l,m)).
10. The prepared single electrode is implanted into the target brain area (Figures 1C and S1, Supporting Information).

### Fabrication of the Silicon‐Based MEAs

The silicon‐based MEAs were fabricated using MEMS technology. The fabrication process involved cleaning Silicon on Insulator (SOI) wafers, Low‐Pressure Chemical Vapor Deposition (LPCVD) deposition of substrate insulation, photolithography, sputtering of Ti/Pt metal layers, lift‐off, Plasma‐Enhanced Chemical Vapor Deposition (PECVD) deposition of top insulation Si_3_N_4_ layer, etching of insulation layer and electrode outlines, etching of the underlying silicon, electrode release, and finally, thorough cleaning of the electrodes.^[^ ^48^, ^54^, ^55^, ^56^, ^57^ ^]^

### Modification of the Tentacle MEAs

A PtNPs/PEDOT:PSS modification approach was employed to achieve a lower SNR and enhance tentacle MEA biocompatibility. This approach was similar to what had been previously reported.^[^ ^57^ ^]^ The PtNPs solution was prepared by mixing 48 mm H_2_PtCl_6_ and 4.2 mm Pb(CH_3_COO)_2_ in a 1:1 ratio. The PEDOT:PSS solution was created by adding 20 mm EDOT to a 0.1 m PSS solution and, by ultrasonication, dispersing the mixture for 30 min. Subsequently, PtNP substrates were electro‐deposited onto the electrodes using a PtNP solution (−1.25 V, 60 s). Then, a PEDOT:PSS layer was electroplated onto the tentacle MEA modified with platinum black (CV, 0–0.95 V, 8 cycles).

Chloroplatinic acid (H_2_PtCl_6_) and lead acetate [Pb(CH_3_COO)_2_] were acquired from Sinopharm Chemical Reagent Co., Ltd. in China. The compound 3,4‐ethoxylenedioxythiophene (EDOT) was sourced from Aladdin Industrial Corporation, China. Poly (sodium‐4‐styrene sulfonate) (PSS) was procured from HEROCHEM, China.

### The Implantation of the Tentacle MEAs

The in vivo performance of tentacle MEAs was assessed through acute and chronic surgery conducted in the CA1 region. However, Young's modulus of tentacle MEA was higher than that of brain tissue. PEG was coated on the implantation needle's surface to address this, enabling the tentacle MEA to be implanted into the brain (Figure 1F,G,H). The coating process was as follows: PEG2000 was melted in a 70 °C water bath, and then the tentacle MEA was dipped into the melted PEG, held for 3 s, and slowly withdrawn. After a few minutes, the PEG solidified, enhancing the hardness of the tentacle MEA implantation needle.

All mice were anesthetized with 0.5–1.5% isoflurane in O_2_ for induction and maintained at 0.6–1% during the later surgery stages. Body temperature was maintained at 37 °C with a thermostatically‐controlled heating pad, and Vaseline was spread on the eyes to prevent dryness and irritation.

The mice's head was secured in a stereotaxic frame, and the skin and connective tissue covering the skull were removed. Four holes were drilled using a high‐speed cranial drill, and skull screws were fitted into these holes. The tentacle MEA was positioned at the bregma as the reference point, and then marking the implantation site for the CA1 region (AP: 2.3, ML: +2, DV: 1.4–1.6 relative to bregma)^[^ ^58^ ^]^ (Figure 3B–D). Subsequently, a 2 × 2 mm square skull was removed at the marked position using a high‐speed cranial drill, and the dura mater was separated with the tip of a syringe needle. A gelatin sponge soaked in physiological saline was placed over the exposed brain tissue to maintain hemostasis and moisture retention.

The tentacle MEA was slowly inserted beneath the skull surface to a depth of 1.45 mm. Ground wires from the tentacle MEA were wound around the skull screws. To secure the implantation needle in place and prevent tentacle MEA detachment, dental cement was spread over the needle, skull screws, and the exposed skull, forming a hemisphere. After surgery, the mice recovered on an electric heating blanket and were individually housed for observation.

### Scenario Setup for the Tentacle MEAs Detecting

To detect spatial cognition neurons in the mice hippocampal CA1 region using the tentacle MEA, a kinematic scenario suitable for mice was constructed (Figure 4A). A circular platform with a diameter of 80 cm and a height of 10 cm above the ground was built. Copper mesh and aluminum foil were sequentially attached above the circular platform and connected to copper mesh on the ground through wires to reduce external noise. A smooth bearing was fixed at the center of the circular platform, connecting a 15 cm upright rod, further connected to a 33 cm crossbar through right‐angle connectors. Subsequently, the crossbar was secured to a headstage fabricated using 3D printing. During the trial, the headstage was worn on the mice's heads to ensure that each lap's running direction and radius remained consistent. Because of the smooth bearing attachment on the central vertical rod, the load on the mouse was minimal, allowing unrestricted free movement. Acrylic walls surrounded the circular platform, and different patterns were applied according to the trial design. The trial included three scenes: continuous black triangles, blue circles, and green rectangles; discrete black triangles, blue circles, and green rectangles; and finally, no stickers (Figure 4B). This setup allowed for comprehensive trials and observation.

### Behavioral Training

After tentacle MEA implantation, the mice were equipped with a headstage, and their heads were fixed to a crossbar in the open field. They were trained for 30 min daily for 14 days until they could comfortably run in the head‐fixed circular open field. Each trial was conducted in a dark cycle. Before the trial, mice were placed in the open field and allowed to adapt for half an hour with the head‐fixed headstage. Mice were then given a rest for half an hour before the commencement of testing.

### Electrophysiological Signal Recording

Electrophysiological signals of hippcampus were recorded using tentacle MEAs through a self‐constructed electrophysiology recording system (AIRCAS‐128, China).^[^ ^59^ ^]^ The system sampled signals at a rate of 30 kHz, the neural spike events characterized by a duration of 3 ms, a baseline cutoff frequency for spike detection set at 200 Hz, and a detection threshold of −30 µV. Neural spikes and LFPs were divided by a highpass filter (>250 Hz) and a low‐pass filter (0–250 Hz), respectively.

### Behavioral Recording and Signal Analysis

An overhead optical camera recorded the running trajectories of mice, and the XY coordinates of the trajectories were marked using the behavioral analysis software (EthoVision XT 15, Noldus, China). Match the recorded electrophysiological signals with the corresponding behavioral locations of the animals. Spike classification/clustering was performed using Offline Sorter. Neurons were segregated based on waveform parameters, such as peak amplitude and principal components. Clusters containing similar valid waveforms were manually defined, ensuring that single units did not exhibit a refractory period <1 ms. Both the spike signals and the video data from the camera were concurrently recorded for comprehensive analysis.

### Soft Boundary Responses Neuron Analysis

Spike data were segmented into small segments on the floor of a maze, 1 cm in width, to create initial maps detailing the number of spikes and their firing positions. A Gaussian kernel function, with a variance of 1 cm, was applied to these raw spikes and occupancy time maps. The spike map was then divided by the occupancy map to produce a smoothed rate map. Each spike position was overlaid onto a Gaussian distribution centered at each recorded trajectory point. The firing field is a contiguous area within a circular open field with an outer diameter of 80 cm and a track width of 5 cm. This field is characterized by a peak firing rate exceeding twice the average firing rate observed in the open field and a peak firing rate higher than 2 Hz. Based on the criteria for single‐neuron spike detection and classification^[^ ^60^, ^61^ ^]^ and 100 shuffles of spikes relative to their firing positions,^[^ ^47^, ^48^ ^]^ 21 groups of soft boundary neurons across seven mice were ultimately identified.

### Analysis of the Spatial Localization Ability of Soft Boundary Neurons

Time Selectivity is defined as:^[^ ^62^ ^]^

(1) $$\mathrm{Time}\;\mathrm{Selectivity}=\frac{\mathrm{Maximum}\;\mathrm{time}\;\mathrm{firing}\;\mathrm{rate}}{\mathrm{Averaged}\;\mathrm{spatial}\;\mathrm{firing}\;\mathrm{rate}}$$

Spatial Selectivity is defined as:^[^ ^62^ ^]^

(2) $$\mathrm{Spatial}\;\mathrm{Selectivity}=\frac{\mathrm{Maximum}\;\mathrm{spatial}\;\mathrm{firing}\;\mathrm{rate}}{\mathrm{Averaged}\;\mathrm{spatial}\;\mathrm{firing}\;\mathrm{rate}}$$

Spatial Information: To evaluate the spatial information in the firing rates of soft boundary neurons, the method utilized to quantify this information was employed, expressed in bits per spike, as follows:^[^ ^63^ ^]^

(3) $$\mathrm{SPI}=\sum_{i}p_{i}\frac{\lambda_{i}}{\lambda }\;\mathrm{lo}g_{2}\frac{\lambda_{i}}{\lambda }$$

where *λ*_i_ represents the average firing rate of a unit in the *i*_th_ bin, *λ*_i_ denotes the general mean firing rate, and *p*_i_ indicates the likelihood of the animal's presence in the *i*_th_ bin (determined by the occupancy time in the *i*_th_ bin divided by the total recording time).

Spatial Coherence: Spatial coherence was estimated as the first‐order spatial autocorrelation of the unsmoothed firing field map, i.e., the mean correlation between the firing rate of each bin and the average firing rate in the 8 adjacent bins.^[^ ^64^ ^]^

Spatial Sparsity: Sparsity index was defined as:^[^ ^62^ ^]^

(4)

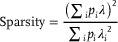

where *λ*_i_ represents the average firing rate of a unit in the *i*_th_ bin, *λ*_i_ denotes the general mean firing rate, and *p*_i_ indicates the likelihood of the animal's presence in the *i*_th_ bin (determined by the occupancy time in the *i*_th_ bin divided by the total recording time).

### Perfusion, HE, and Fluorescent Staining

After signal detection, the mice underwent sequential cardiac perfusion with physiological saline and 4% paraformaldehyde. Subsequently, the mouse brain was taken out and immersed first in a 20% sucrose solution until it settled and then in a 30% sucrose solution until it settled. Finally, the brain was sectioned into 40 µm slices using a freezing microtome, and the needle implantation site was examined under a microscope.

For mice requiring HE and immunofluorescence staining, the mice were perfused with saline and the brain was harvested. The brain tissue was directly embedded at −20 °C. Ten micometer brain tissue sections were prepared for separate HE and fluorescent staining.

For HE staining, first the sections were fixed with 4% acetone for 15 min. Then, the sections were dewaxed to water: immersed in xylene I and II for 5 min each, followed by 100% ethanol I, 100% ethanol II, 95% ethanol, 85% ethanol, and 75% ethanol for 2 min each, and rinsed in tap water for 4 min. The sections were stained with hematoxylin for 1 min, then rinsed in tap water for 2 min. Differentiated in 1% hydrochloric acid in ethanol for 5 s and washed in tap water for 4 min. Stained with eosin for 1 min, followed by a 5 s water rinse. The sections were dehydrated and cleared in 85% ethanol, 95% ethanol, 100% ethanol I, and 100% ethanol II for 2 min each, followed by xylene I and II for 5 min each. Finally, the sections with neutral resin were mounted.

For fluorescent staining, first 8% PFA was fixed for 10 min and washed three times with PBS for 5 min each. Next, it was incubated in 1 mg mL^−1^ quenching solution for 7 min to quench tissue background fluorescence. Then, it was washed three times with PBS for 5 min each. Then, the brain sections were blocked and permeabilized with 5% BSA and 10% Triton, incubated with primary antibodies, Rabbit – TMEM119 and Mouse – GFAP, overnight at 4 °C, followed with secondary antibodies, directly labeled antibodies, and directly labeled dyes, Rabbit‐STAR Red, Mouse‐STAR Red, TUBBIII, Hochest‐405, for 1 h at room temperature. The sections were post‐fixed and mounted, and then incubated in 4% PFA solution at room temperature for 10 min, and the sections were washed three times with sterile water for 5 min each. The sections were mounted using a medium compatible with confocal microscopy. The average immunofluorescence density was analyzed using Image J software.

### Statistical Analysis

All quantitative data were presented as the mean ± standard deviation (mean ± SD). The Student's t‐test with two‐tailed confidence intervals was utilized to compare the two groups using Graph Pad Prism 8.0 (GraphPad Software, Inc., CA, USA) and Microcal Origin Pro 8.5.1 (Origin Lab. Corp., Northampton, MA, USA). *p*‐Value < 0.05 was considered statistically significant.

## Conflict of Interest

The authors declare no conflict of interest.

## Author Contributions

S.L. performed formal analysis and wrote the original draft. F.M., Z.X., Y.W., G.Y., Y.D., Y.L., and M.L. performed investigations. M.H., L.J., and W.X. performed validation. J.L., J.L., and M.W. performed formal analysis. Y.S., Y.W., and X.C. performed project administration, acquired funding, and supervised the study.

## Supporting information

Supporting Information

## Data Availability

The data that support the findings of this study are available from the corresponding author upon reasonable request.

## References

[1] J. O'Keefe , N. Burgess , Nature 1996, 381, 425. 8632799 10.1038/381425a0

[2] J. O'Keefe , Exp. Neurol. 1976, 51, 78. 1261644 10.1016/0014-4886(76)90055-8

[3] J. O'Keefe , D. H. Conway , Exp Brain Res 1978, 31. 10.1007/BF00239813 658182

[4] K. J. Jeffery , M. I. Anderson , Hippocampus 2003, 13, 868. 14620882 10.1002/hipo.10162

[5] C. Dong , A. D. Madar , M. E. J. Sheffield , Nat. Commun. 2021, 12, 2977. 34016996 10.1038/s41467-021-23260-3 PMC8137926

[6] F. Sharif , B. Tayebi , G. Buzsáki , S. Royer , A. Fernandez‐Ruiz , Neuron 2021, 109, 363. 33217328 10.1016/j.neuron.2020.10.034 PMC7856084

[7] S. L. Pashkovski , G. Iurilli , D. Brann , D. Chicharro , K. Drummey , K. M. Franks , S. Panzeri , S. R. Datta , Nature 2020, 583, 253. 32612230 10.1038/s41586-020-2451-1 PMC7450987

[8] R. P. Jayakumar , M. S. Madhav , F. Savelli , H. T. Blair , N. J. Cowan , J. J. Knierim , Nature 2019, 566, 533. 30742074 10.1038/s41586-019-0939-3 PMC6629428

[9] A. D. Redish , Beyond the Cognitive Map: From Place Cells to Episodic Memory, MIT Press, Cambridge, MA 1999.

[10] L. McNaughton , L. L. Chen , J. Markus , J. Cogn. Neurosci. 1991, 3, 190. 23972093 10.1162/jocn.1991.3.2.190

[11] J. O'Keefe , N. Burgess , J. G. Donnett , K. J. Jeffery , E. A. Maguire , Phil. Trans. R. Soc. Lond. B 1998, 353, 1333. 9770226 10.1098/rstb.1998.0287 PMC1692339

[12] X. Tang , H. Shen , S. Zhao , N. Li , J. Liu , Nat. Electron. 2023, 6, 109.

[13] R. Chen , A. Canales , P. Anikeeva , Nat. Rev. Mater. 2017, 2, 16093. 31448131 10.1038/natrevmats.2016.93 PMC6707077

[14] V. S. Polikov , P. A. Tresco , W. M. Reichert , J. Neurosci. Methods 2005, 148, 1. 16198003 10.1016/j.jneumeth.2005.08.015

[15] J. W. Salatino , K. A. Ludwig , T. D. Y. Kozai , E. K. Purcell , Nat. Biomed. Eng. 2017, 1, 862. 30505625 10.1038/s41551-017-0154-1 PMC6261524

[16] N. Adly , S. Weidlich , S. Seyock , F. Brings , A. Yakushenko , A. Offenhäusser , B. Wolfrum , npj Flex Electron 2018, 2, 15.

[17] W. J. Tyler , Nat. Rev. Neurosci. 2012, 13, 867. 23165263 10.1038/nrn3383

[18] M. Yan , L. Wang , Y. Wu , X. Liao , C. Zhong , L. Wang , Y. Lu , ACS Appl. Mater. Interfaces 2023, 15, 41310. 37590473 10.1021/acsami.3c07189

[19] Q. Liang , X. Xia , X. Sun , D. Yu , X. Huang , G. Han , S. M. Mugo , W. Chen , Q. Zhang , Adv. Sci. 2022, 9, 2201059. 10.1002/advs.202201059 PMC9165511 35362243

[20] H. Zheng , Z. Zhang , S. Jiang , B. Yan , X. Shi , Y. Xie , X. Huang , Z. Yu , H. Liu , S. Weng , A. Nurmikko , Y. Zhang , H. Peng , W. Xu , J. Zhang , Nat. Commun. 2019, 10, 2790. 31243276 10.1038/s41467-019-10418-3 PMC6594927

[21] A. Stiller , J. Usoro , C. Frewin , V. Danda , M. Ecker , A. Joshi‐Imre , K. Musselman , W. Voit , R. Modi , J. Pancrazio , B. Black , Micromachines 2018, 9, 500. 30424433 10.3390/mi9100500 PMC6215160

[22] A. Stiller , J. Usoro , J. Lawson , B. Araya , M. González‐González , V. Danda , W. Voit , B. Black , J. Pancrazio , Micromachines 2020, 11, 619. 32630553 10.3390/mi11060619 PMC7344527

[23] A. Zátonyi , G. Orbán , R. Modi , G. Márton , D. Meszéna , I. Ulbert , A. Pongrácz , M. Ecker , W. E. Voit , A. Joshi‐Imre , Z. Fekete , Sci. Rep. 2019, 9, 2321. 30787389 10.1038/s41598-019-39835-6 PMC6382803

[24] J. M. Seo , S. J. Kim , H. Chung , E. T. Kim , H. G. Yu , Y. S. Yu , Mater. Sci. Eng., C 2004, 24, 185.

[25] S. H. Cho , H. M. Lu , L. Cauller , M. I. Romero‐Ortega , J. B. Lee , G. A. Hughes , IEEE Sensors J 2008, 8, 1830.

[26] A. Imai , S. Takahashi , S. Furubayashi , Y. Mizuno , M. Sonoda , T. Miyazaki , E. Miyashita , T. Fujie , Adv. Mater. Technol. 2023, 8, 2300300.

[27] J. M. Kim , C. Im , W. Lee , Polymers 2017, 9, 690. 30965988 10.3390/polym9120690 PMC6418796

[28] S. Guan , H. Tian , Y. Yang , M. Liu , J. Ding , J. Wang , Y. Fang , Nat. Protoc. 2023, 18, 1712. 37248393 10.1038/s41596-023-00824-9

[29] H. Xu , A. Weltman , M. C. Hsiao , K. Scholten , E. Meng , T. W. Berger , D. Song , in 2016 38th Annual Int. Conf. of the IEEE Engineering in Medicine and Biology Society (EMBC) , IEEE, Orlando, FL 2016, pp. 2806–2809. 10.1109/EMBC.2016.7591313 28268901

[30] H. Xu , K. Scholten , Z. Li , E. Meng , D. Song , in 2023 45th Annual Int. Conf. of the IEEE Engineering in Medicine & Biology Society (EMBC) , IEEE, Sydney, Australia, 2023, pp. 1–4. 10.1109/EMBC40787.2023.10340804 38083000

[31] X. Wang , A. W. Hirschberg , H. Xu , Z. Slingsby‐Smith , A. Lecomte , K. Scholten , D. Song , E. Meng , J. Microelectromech. Syst. 2020, 29, 499. 35663261 10.1109/jmems.2020.3000235 PMC9164222

[32] H. Xu , A. W. Hirschberg , K. Scholten , T. W. Berger , D. Song , E. Meng , J. Neural Eng. 2018, 15, 016017. 29044049 10.1088/1741-2552/aa9451 PMC5792195

[33] H. Xu , A. W. Hirschberg , K. Scholten , E. Meng , T. W. Berger , D. Song , in 2018 40th Annual Int. Conf. of the IEEE Engineering in Medicine and Biology Society (EMBC) , IEEE, Honolulu, HI, 2018, pp. 4599–4602. 10.1109/EMBC.2018.8513202 PMC7153783 30441376

[34] H. Xu , A. Weltman , K. Scholten , E. Meng , T. W. Berger , D. Song , in 2017 39th Annual Int. Conf. of the IEEE Engineering in Medicine and Biology Society (EMBC) , IEEE, Jeju, Korea (South), 2017, pp. 1716–1719. 10.1109/EMBC.2017.8037173 29060217

[35] H. Xu , M. Hsiao , D. Song , T. W. Berger , in 2014 36th Annual Int. Conf. of the IEEE Engineering in Medicine and Biology Society , IEEE, Chicago, IL, 2014, pp. 4876–4879. 10.1109/EMBC.2014.6944716 25571084

[36] C. Cointe , A. Laborde , L. G. Nowak , D. N. Arvanitis , D. Bourrier , C. Bergaud , A. Maziz , Microsyst. Nanoeng. 2022, 8, 21. 35251687 10.1038/s41378-022-00353-7 PMC8847482

[37] J. T. W. Kuo , B. J. Kim , S. A. Hara , C. D. Lee , C. A. Gutierrez , T. Q. Hoang , E. Meng , Lab Chip 2013, 13, 554. 23160191 10.1039/c2lc40935f

[38] P. Fan , Y. Wang , Y. Dai , L. Jing , W. Liang , B. Lu , G. Yang , Y. Song , Y. Wu , X. Cai , Sens. Actuators, B 2023, 390, 133990.

[39] F. Mo , Z. Xu , G. Yang , P. Fan , Y. Wang , B. Lu , J. Liu , M. Wang , L. Jing , W. Xu , M. Li , J. Shan , Y. Song , X. Cai , Biosens. Bioelectron. 2022, 217, 114726. 36174358 10.1016/j.bios.2022.114726

[40] E. Castagnola , E. M. Robbins , D. D. Krahe , B. Wu , M. Y. Pwint , Q. Cao , X. T. Cui , Biosens. Bioelectron. 2023, 230, 115242. 36989659 10.1016/j.bios.2023.115242 PMC10101938

[41] R. Cornuéjols , A. Albon , S. Joshi , J. A. Taylor , M. Baca , S. Drakopoulou , T. Rinaldi Barkat , C. Bernard , S. Rezaei‐Mazinani , ACS Appl. Mater. Interfaces 2023, 15, 22854. 37141163 10.1021/acsami.3c00553 PMC10197075

[42] D. Khodagholy , J. N. Gelinas , T. Thesen , W. Doyle , O. Devinsky , G. G. Malliaras , G. Buzsáki , Nat. Neurosci. 2015, 18, 310. 25531570 10.1038/nn.3905 PMC4308485

[43] A. Fendyur , N. Mazurski , J. Shappir , M. E. Spira , Front. Neuroeng 2011, 4, 14. 22163219 10.3389/fneng.2011.00014 PMC3233721

[44] C. Xie , L. Hanson , W. Xie , Z. Lin , B. Cui , Y. Cui , Nano Lett. 2010, 10, 4020. 20815404 10.1021/nl101950x PMC2955158

[45] L. Muzio , A. Viotti , G. Martino , Front. Neurosci. 2021, 15, 742065. 34630027 10.3389/fnins.2021.742065 PMC8497816

[46] F. Giovannoni , F. J. Quintana , Trends Immunol. 2020, 41, 805. 32800705 10.1016/j.it.2020.07.007 PMC8284746

[47] F. Sargolini , M. Fyhn , T. Hafting , B. L. McNaughton , M. P. Witter , M. B. Moser , E. I. Moser , Science 2006, 312, 758. 16675704 10.1126/science.1125572

[48] Z. Xu , F. Mo , G. Yang , P. Fan , B. Lu , W. Liang , F. Kong , L. Jing , W. Xu , J. Liu , M. Wang , Y. Wu , X. Cai , Research 2023, 6, 0229. 37719050 10.34133/research.0229 PMC10503993

[49] C. H. Wang , J. D. Monaco , J. J. Knierim , Curr. Biol. 2020, 30, 1397. 32109393 10.1016/j.cub.2020.01.083 PMC7259364

[50] N. Nyberg , É. Duvelle , C. Barry , H. J. Spiers , Neuron 2022, 110, 394. 35032426 10.1016/j.neuron.2021.12.012

[51] T. Geiller , M. Fattahi , J. S. Choi , S. Royer , Nat. Commun. 2017, 8, 14531. 28218283 10.1038/ncomms14531 PMC5321734

[52] P. A. Hetherington , M. L. Shapiro , Behav. Neurosci. 1997, 111, 20. 9109621 10.1037//0735-7044.111.1.20

[53] M. H. Plitt , L. M. Giocomo , Nat. Neurosci. 2021, 24, 705. 33753945 10.1038/s41593-021-00816-6 PMC8893323

[54] Z. Xu , F. Mo , G. Yang , P. Fan , Y. Wang , B. Lu , J. Xie , Y. Dai , Y. Song , E. He , S. Xu , J. Liu , M. Wang , X. Cai , Microsyst. Nanoeng. 2022, 8, 104. 36124081 10.1038/s41378-022-00436-5 PMC9481550

[55] G. Xiao , S. Xu , Y. Song , Y. Zhang , Z. Li , F. Gao , J. Xie , L. Sha , Q. Xu , Y. Shen , X. Cai , Sens. Actuators, B 2019, 288, 601.

[56] G. Xiao , Y. Song , Y. Zhang , Y. Xing , H. Zhao , J. Xie , S. Xu , F. Gao , M. Wang , G. Xing , X. Cai , ACS Sens. 2019, 4, 1992. 31272150 10.1021/acssensors.9b00182

[57] G. Yang , Y. Wang , Z. Xu , X. Zhang , W. Ruan , F. Mo , B. Lu , P. Fan , Y. Dai , E. He , Y. Song , C. Wang , J. Liu , X. Cai , Biosensors 2023, 13, 496. 37232857 10.3390/bios13050496 PMC10216047

[58] K. B. J. Franklin , G. Paxinos , Paxinos and Franklin's the Mouse Brain in Stereotaxic Coordinates, Compact: The Coronal Plates and Diagrams, Academic Press, Cambridge, MA 2019.

[59] S. Xu , Y. Zhang , S. Zhang , G. Xiao , M. Wang , Y. Song , F. Gao , Z. Li , P. Zhuang , P. Chan , G. Tao , F. Yue , X. Cai , J. Neurosci. Methods 2018, 304, 83. 29698630 10.1016/j.jneumeth.2018.04.015

[60] K. D. Harris , D. A. Henze , J. Csicsvari , H. Hirase , G. Buzsáki , J. Neurophysiol. 2000, 84, 401. 10899214 10.1152/jn.2000.84.1.401

[61] S. N. Kadir , D. F. M. Goodman , K. D. Harris , Neural Computation 2014, 26, 2379. 25149694 10.1162/NECO_a_00661 PMC4298163

[62] W. E. Skaggs , B. L. McNaughton , M. A. Wilson , C. A. Barnes , Hippocampus 1996, 6, 149. 8797016 10.1002/(SICI)1098-1063(1996)6:2<149::AID-HIPO6>3.0.CO;2-K

[63] W. E. Skaggs , B. L. McNaughton , K. M. Gothard , Neural Inf. Process Syst 1993, 1030.

[64] L. Lu , K. M. Igarashi , M. P. Witter , E. I. Moser , M. B. Moser , Neuron 2015, 87, 1078. 26298277 10.1016/j.neuron.2015.07.007

