# Supplementary material for: Tentacle Microelectrode Arrays Uncover Soft Boundary Neurons in Hippocampal CA1
Source: Adv Sci (Weinh). 2024 Jun 3;11(29):2401670. doi: 10.1002/advs.202401670 (PMC11304256; doi:10.1002/advs.202401670)
Supplement: Supplementary file 1 — Supporting Information [file ADVS-11-2401670-s001.docx]

Supporting Information

Tentacle Microelectrode Arrays Uncover Soft Boundary Neurons in Hippocampal CA1

*Shiya Lv ^1,2^, Fan Mo^1,2^, Zhaojie Xu^1,2^, Yu Wang^1,2^, Gucheng Yang^1,2^, Meiqi Han^1,2^, Luyi Jing^1,2^, Wei Xu^1,2^, Yiming Duan^1,2^, Yaoyao Liu^1,2^, Ming Li^1,2^, Juntao Liu^1,2^, Jinping Luo^1,2^, Mixia Wang^1,2^, Yilin Song^1,2^*, Yirong Wu^1,2^*, Xinxia Cai^1,2^**

1. State Key Laboratory of Transducer Technology, Aerospace Information Research Institute, Chinese Academy of Sciences, Beijing 100190, China.

2. University of Chinese Academy of Sciences, Beijing 100049, China.

*Corresponding author: Xinxia Cai ([xxcai@mail.ie.ac.cn](mailto:xxcai@mail.ie.ac.cn)); Yirong Wu ([wyr@mail.ie.ac.cn](mailto:wyr@mail.ie.ac.cn)); Yilin Song(ylsong@mail.ie.ac.cn).


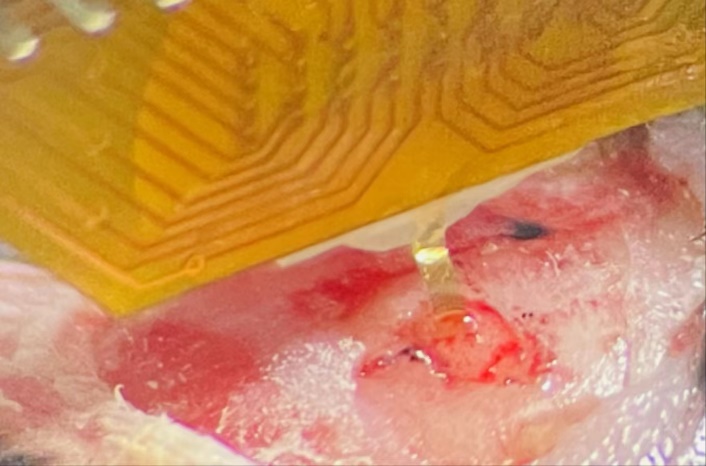


Figure S1. Tentacle MEA implantation completed. The yellow device is a polyimide-based flexible printed circuit electrode connector board. It connects the tentacle microelectrode to the backend signal processing circuit (eg. headstage).


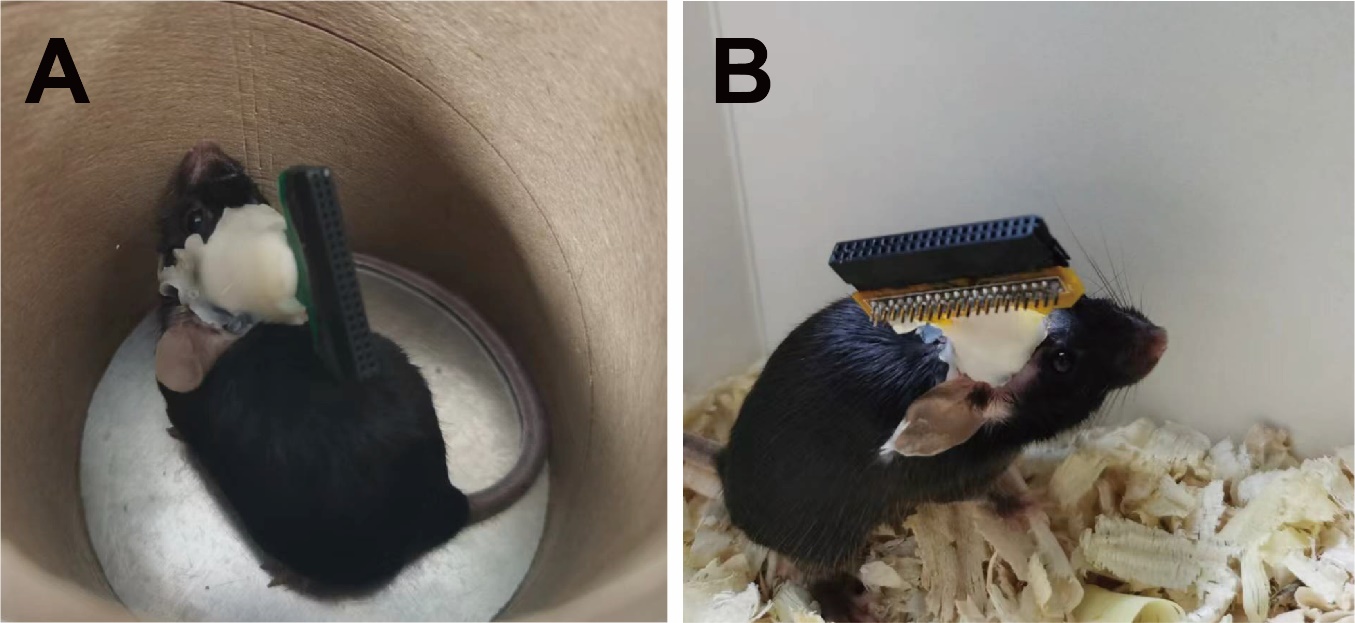


Figure S2. Mice with implanted tentacle MEAs.

A), A mouse implanted with the 32-channel silicon-based MEA.

B), A mouse implanted with the 32-channel tentacle MEA.


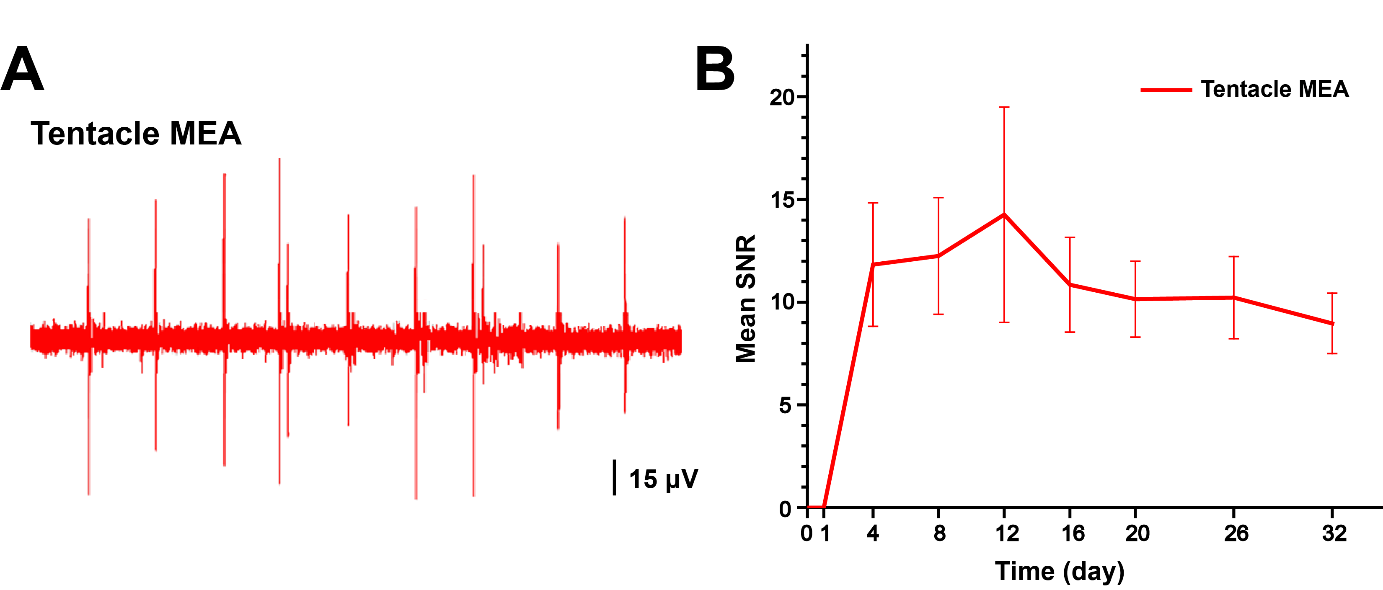


Figure S3. Signal-to-noise ratio (SNR) of spike recording.

A), Typical spike SNR display.

B), Changes in the mean SNR of electrophysiological data were recorded by the tentacle MEA.
